# Impaired nuclear factor erythroid 2-related factor 2 expression increases apoptosis of airway epithelial cells in patients with chronic obstructive pulmonary disease due to cigarette smoking

**DOI:** 10.1186/s12890-016-0189-1

**Published:** 2016-02-09

**Authors:** Kazuhiro Yamada, Kazuhisa Asai, Fumihiro Nagayasu, Kanako Sato, Naoki Ijiri, Naoko Yoshii, Yumiko Imahashi, Tetsuya Watanabe, Yoshihiro Tochino, Hiroshi Kanazawa, Kazuto Hirata

**Affiliations:** Department of Respiratory Medicine, Graduate School of Medicine, Osaka City University, 1-4-3, Asahi-machi, Abeno-ku, Osaka 545-8585 Japan

**Keywords:** Chronic obstructive pulmonary disease (COPD), Nuclear factor erythroid 2-related factor 2 (Nrf2), Oxidative stress, Apoptosis, Epithelial cell, N-acetyl cysteine (NAC), Cigarette smoke extract (CSE), Time-lapse line cell imaging assay

## Abstract

**Background:**

Cigarette smoking-induced oxidative stress is known to be a key mechanism in COPD pathogenesis. Nuclear factor erythroid 2-related factor 2 (Nrf2) is a central transcription factor that regulates the antioxidant defense system. The aim of this study was to compare Nrf2 expression in COPD subjects and control subjects, and to determine the role of Nrf2 in protecting against oxidative stress-induced apoptosis.

**Methods:**

We enrolled 8 COPD subjects and 7 control subjects in this study. We performed bronchial brushing by bronchoscopy and obtained bronchial epithelial cells from the airways. Nrf2 expression in bronchial epithelial cells was evaluated by real-time PCR and Western blotting. We examined the effect of 10 or 15 % cigarette smoke extract (CSE) induced A549 cells apoptosis using a time-lapse cell imaging assay with caspase-3/7 activation detecting reagent and performed Terminal deoxynucleotidyltransferase-mediated dUTP nick end labelling assay for confirming A549 cells apoptosis. We also examined the effects of Nrf2 knockdown and, 0.1, 0.5, and 1.0 mM N-acetyl cysteine on CSE-induced apoptosis. Statistical analyses were performed using t-test, paired t-test or an analysis of variance followed by the Tukey-Kramer method.

**Results:**

Nrf2 mRNA expression in COPD subjects was significantly lower than that in control subjects and Nrf2 mRNA were negatively correlated with pack year. Nrf2 protein in COPD subjects was significantly lower than that in control subjects. CSE-induced A549 cells apoptosis was increased in a time-, concentration-dependent manner, and was significantly increased by Nrf2 knockdown. N-acetyl cysteine significantly ameliorated CSE-induced apoptosis.

**Conclusions:**

Nrf2 expression was lower in COPD patients than in control subjects. Nrf2 might have a protective role against apoptosis caused by CSE-induced oxidative stress. These results suggest an involvement of Nrf2 in COPD and administration of antioxidants to patients with COPD might be a basic therapeutic option.

## Background

Chronic obstructive pulmonary disease (COPD) is projected to become the third most prevalent cause of mortality in the world by 2020 [1], and it is a major concern in terms of health economics. Although COPD is defined and categorized by limitations in airflow, it is not a single disease. Pathological changes in COPD include changes in the central or distal airways. The former is considered chronic bronchitis, and this disease is accompanied by an influx of inflammatory cells in the mucosa and airway remodeling, such as epithelial metaplasia and mucus hypersecretion due to hyperplasia of subepithelial mucus glands or goblet cells. The latter is considered emphysema, and alveolar wall destruction and disrupted alveolar attachments to small airway walls are seen with this condition. Inhalation of noxious gas is known to cause COPD, and cigarette smoking, specifically, is recognized as the major cause of COPD. Cigarette smoke has a gas component and a small particulate matter component of less than 5 μm, and with inhaled cigarette smoke, these components will reach the small airways and evoke pathological changes in the alveolar walls. Oxidative stress is now recognized as a major predisposing factor in the pathogenesis of COPD. Cigarette smoke contains reactive oxygen species (ROS) and each puff of tobacco smoke contains approximately 1x10^17^ oxidant molecules [2]. ROS can cause oxidative stress and play a major role in cell apoptosis through injury induced by cigarette smoke [3]. Excessive loss of alveolar epithelial cells and endothelial cells by apoptosis has been postulated to lead to the destruction of lung tissue and emphysema.

Nuclear factor erythroid 2-related factor 2 (Nrf2), also known as NFE2L2, is ubiquitously expressed at high concentrations in various human organs including the lungs. Although Nrf2 exists in the cytoplasm with a cluster of proteins that degrade it quickly under normal conditions, following oxidative stress, Nrf2 reaches the nucleus of a cell and binds to the antioxidant response element (ARE) in the upstream promoter region of antioxidant genes and initiates transcription of these genes and expression of these proteins [4]. Nrf2 is a central transcription factor that regulates the antioxidant defense system and acts as a modifier of several lung diseases associated with oxidative stress and inflammation. Murine studies report that the knockdown of Nrf2 made mice highly susceptible to cigarette smoke-induced lung injury [5, 6]. Moreover, Nrf2 activation in macrophages reduced oxidative stress [7]. These studies suggest that there is a clear link between defects in the lung antioxidant defense system regulated by Nrf2 and oxidative stress-related lung diseases [8]. However, in humans, no previous studies have addressed the relationship of Nrf2 expression to oxidative stress and COPD.

In this study, we determined the level of Nrf2 in the bronchial epithelial cells by RT-PCR and Western blotting, and alveolar epithelial cells by immunohistochemistry in COPD subjects. We also examined the effect of antioxidants and Nrf2 expression on CSE-induced apoptosis in vitro.

## Methods

### Human bronchial epithelial cells

Study subjects were patients scheduled for bronchoscopies for small nodules or a small amount of bloody sputum in an outpatient clinic at Osaka City University. We performed bronchial brushing from the normal side by bronchoscopy and obtained bronchial epithelial cells from the airways of 8 COPD subjects and 7 healthy control (CTL) subjects. COPD is defined by The Global Initiative for Chronic Obstructive Lung Disease (GOLD) guidelines as a post-bronchodilator FEV1 % (FEV1/FVC) of <70 % [9]. This study was approved by the ethics committee of Osaka City University Hospital (approval number 1819) and all patients gave written informed consents for their participation.

### Real-time PCR

Total RNA was extracted using 500 μL of TRIzol (Life Technologies Japan, Tokyo, Japan) from cells obtained by bronchoscopic bronchial brushing. Reverse transcription of 1 μg of total RNA was performed using Ready-To-Go T-Primed First-Strand Kit (GE Healthcare Japan, Tokyo, JAPAN) . One μL of sample cDNA was added to a PCR mixture containing 1 μL of primers targeting Nrf2 and GAPDH by TaqMan^®^ Gene Expression Assays (Life Technologies Japan, Tokyo, Japan), 10 μL of 2× Universal PCR Master Mix (Life Technologies Japan, Tokyo, Japan), and 8 μL of DEPC Water. Quantitative real-time PCR was performed using an Applied Biosystems 7500 Real-Time PCR System (Life Technologies Japan, Tokyo, Japan). The PCR conditions used in all reactions were: 10 min at 95 °C, followed by 40 two-step cycles (95 °C for 15 s and 60 °C for 45 s). The relative levels of Nrf2 mRNA were normalized to GAPDH mRNA using the ΔΔCt method.

### Western blotting and detection

Cells obtained by bronchoscopic bronchial brushing were suspended in 4 ml of PBS. The PBS were centrifuged at 10,000 × *g* at 4 °C for 10 min. Supernatants were discarded and pellet was dissolved with 100 μL of cell lysis buffer (Cell Signaling Technology, Danvers, MA, USA) The concentrations of the resulting protein extracts were measured using a BCA Protein Assay Kit (Pierce, Waltham, MA, USA). Ten μg of each total protein lysate was subjected to sodium dodecyl sulfate polyacrylamide gel electrophoresis by using Mini-PROTEAN^®^ TGX™ Precast Protein Gels (4561023, Bio-Rad, Hercules, California, USA). The membranes were incubated at 4 °C overnight with primary antibodies against Nrf2 (1:250, ab137550, Abcam, Tokyo, Japan) or β-actin (1:5000, sc-130656, Santa Cruz Biotechnology, Santa Cruz, CA, USA) and were then incubated with secondary antibodies against rabbit IgG produced in goat (sc-2004, Santa Cruz Biotechnology) for 2 h at room temperature. The densitometry to measure protein level in Nrf2 and β-actin was performed by using LAS4000 (Fuji Imaging, Tokyo, Japan).

### Immunohistochemistry

Four COPD and four control subjects formalin-fixed, paraffin-embedded lung sections (4 μm thick) were obtained from tissue bank of Osaka City University. For immunohistochemical staining of Nrf2, the usual avidin-biotin complex (ABC) method was employed. Briefly, after deparaffinized, the slides were washed with PBS. The slides were permeabilized with 0.2 % triton (Sigma Aldrich, St.Louis, MO, USA) in PBS and treated with 5 % hydrogen peroxide in PBS for 30 min at room temperature. The sections were then blocked with 5 % goat serum for 30 min, washed with PBS and incubated overnight with the Nrf2 primary antibody (Santa Cruz Biothechnology, Santa Cruz, CA, USA) at a dilution of 1:100 at 4 °C. The Vectastain ABC elite kit (Vector Laboratories, Burlingame, CA, USA) was used to detect the resulting immune complexes following the manufacturer’s instruction. The peroxidase activity was visualized by using DAB Substrate Kit (SK-4100, Vector Laboratories, Burlingame, CA, USA). The slides were then counterstained with hematoxylin and mounted. Negative controls were performed using nonimmune serum instead of the primary antibody to exclude nonspecific staining. For evaluation of immunoperoxidase staining, two observers blind to clinical information counted immunoperoxidase staining positive alveolar epithelial cells in four non-overlapping fields. Total alveolar septal length of each field was measured by using Image J software (National Institutes of Health, Bethesda, Maryland, USA) and immunoperoxidase positivity of alveolar epithelial cells were adjusted by total alveolar septum length. We calculated the ratio: positive cell number/alveolar septal length.

### Preparations of cigarette smoke extract (CSE)

To mimic cigarette smoking-induced epithelial cell damage, we employed the gas phase of CSE in an in vitro study. CSE was prepared as previously described [10]. Briefly, 40 Marlboro cigarettes (Philip Morris Japan, Tokyo, Japan) were mounted for burning, and the main stream of the smoke was aspirated at a flow rate of 1.05 l/min, which was strictly regulated by the KOFLOC^TM^ Mass Flow Controller (model 8500 series: Kojima Instruments Inc., Kyoto, Japan) and passed through a Cambridge glass fiber filter (Heinr, Borgwaldt GmbH, Hamburg, Germany) to remove the tar phase of the smoke and nicotine. The remaining gas phase of the smoke was bubbled into 10 ml of PBS at 25 °C. The medium was filtered with a 0.22 μm Millex filter (Merck Millipore, Billerica, MA, USA). This solution was defined as 100 % CSE. The CSE was stored at −80 °C before use. CSE was diluted with PBS or cell culture medium for in vitro experiments.

### Exhaled breath condensate (EBC)

To examine the oxidative milieu in smokers’ airways, EBC was collected using ECoScreen^®^ (Eric Jaeger, Würzburg, Germany). Fifteen minutes of oral EBCs were collected before and after cigarette smoking in healthy smoking volunteers without nose clips. The EBC pH was an easily measurable surrogate marker of oxidative stress in the airway [11] that was measured immediately after collection using a pH analyzer (Horiba, Ltd, Kyoto, Japan).

### A549 cell culture

Cigarette smoke induces alveolar cell apoptosis leading to alveolar wall destruction and emphysema. Therefore, we employed A549 cells derived from adenocarcinomic human alveolar basal epithelial cells for in vitro experiments. A549 cells were obtained from American Type Culture Collection and was grown in DMEM/F12 medium (Lonza Japan, Tokyo, Japan) supplemented with 10 % fetal bovine serum (FBS) on 75 cm^2^ cell flasks. Cells were grown to >80 % confluence and passaged to a 96-well plate (Corning 3596, Sigma-Aldrich Japan, Tokyo, Japan) at a density of 1.67 × 10^3^ cells per well in triplicate, such that the next day, cells were approximately 10–20 % confluent. For the time-lapse cell imaging assay, treatment and pretreatment media were prepared as follows. We prepared optimal concentrations of CSE determined by EBC pH and 100 μM H_2_O_2_ as a positive control for cell stimulation in bronchial epithelial growth medium (BEGM) (Lonza Japan, Tokyo, Japan). For pretreatment, we prepared the control medium, 0.1, 0.5, and 1.0 mM N-acetyl cysteine (NAC) (Sigma-Aldrich Japan, Tokyo, Japan), 0.1, 1.0, and 5.0 μg/ml anti-TNF-α antibody (mouse monoclonal IgG_1_ clone: R&D Systems, Inc., Minneapolis, MN, USA) in BEGM.

### Time-lapse cell imaging assay for apoptosis

To examine oxidative stress-induced epithelial cell apoptosis and the protective effect of the antioxidant over time, a time-lapse cell imaging assay (IncuCyte ZOOM^®^ system with Cell Player™ 96-Well Kinetic Caspase-3/7 reagent: Essen BioScience, Ltd., Tokyo, Japan) was performed. Caspase-3/7 reagent coupled the activated caspase-3/7 recognition motif (DEVD) to NucView™488, a DNA intercalating dye. When added to tissue culture medium, this inert, non-fluorescent substrate crosses the cell membrane where it is cleaved by activated caspase-3/7, resulting in the release of the DNA dye and green fluorescent staining of nuclear DNA. Kinetic activation of caspase-3/7 can be monitored morphologically using live cell imaging, and quantified using the IncuCyte ZOOM^®^ Basic Analyzer or the IncuCyte™ FLR object counting algorithm. Caspase-3/7 reagent was diluted and added to each well for a final concentration of 5 μM in each pretreatment media. The 96-well plate was put into a microplate tray in IncuCyte ZOOM. The previously described pretreatment and treatment media were added to each well. The IncuCyte ZOOM acquired phase-contrast images at 3-h intervals over a period of 12 h, and calculated the mean bright green cell count (the Caspase-3/7 positive cell number) from non-overlapping fluorescent images of each well automatically [12]. At the end of the experiment, Vybrant DyeCycle Green (VDCG: Life Technologies Japan, Tokyo, Japan), a cell-permeant DNA-binding fluorophore, was added and incubated for 1 h to provide an absolute (endpoint) nuclear cell count (the total cell number). We defined the apoptotic index according to the following equation:

Apoptotic index = Caspase-3/7-positive cell number/Total cell number

### Nrf2 mRNA knockdown by siRNA

To examine the protective effect of Nrf2 on oxidative stress-induced epithelial cell apoptosis over time, A549 cells were pre-treated with Silencer^®^ select siRNA against Nrf2 (Life Technologies Japan, Tokyo, Japan) and IncuCyte ZOOM system and Cell Player™ 96-Well Kinetic Caspase-3/7 reagent experiments were repeated. Silencer^®^ Negative Control siRNA (Life Technologies Japan, Tokyo, JAPAN) was used as a negative control. A549 cells were transfected with the siRNA complex with Lipofectamine 2000 (Life Technologies Japan, Tokyo, Japan) according to the manufacturer’s recommendations. The knockdown efficiency of Nrf2 was quantified by RT-PCR. The A549 cells were seeded in a 96-well plate at a density of 1.67 × 10^3^ cells per well in triplicate. On the next day, cells were transfected with the siRNA complex. Twenty-four hour later, the cells were treated with 10 % CSE and examined the apoptotic index using IncuCyte ZOOM.

### Terminal deoxynucleotidyltransferase-mediated dUTP nick end labelling assay (TUNEL) and DAPI staining

To confirm CSE-induced apoptosis, not just Caspase-3/7 activation, TUNEL staining (Roche Diagnostics K.K., Tokyo, Japan) was performed. A549 cells were grown on culture slides and fixed in methanol and acetone. Then, slides were incubated for 1 h at 37 °C in the presence of terminal deoxynucleotidyl transferase (TdT). In the negative control, no TdT was added. In addition, after cells were washed with PBS, cells were stained with 2 μg/ml DAPI for 15 min. Slides were observed with a fluorescent microscope at 475–495 nm for TUNEL and 330–385 nm for DAPI at × 400 magnification.

### Statistical analysis

Statistical analyses were performed using JMP version 10.0.0 software for Windows (SAS Institute Inc., Cary, NC, USA). To compare 2 groups, a t-test or paired t-test was performed. To compare more than 3 groups, an analysis of variance (ANOVA) followed by the Tukey–Kramer method were performed. In all statistical analyses, *p*-values < 0.05 were considered significant.

## Results

### Nrf2 mRNA and protein level decreased in COPD subjects

**Fig. 1:**
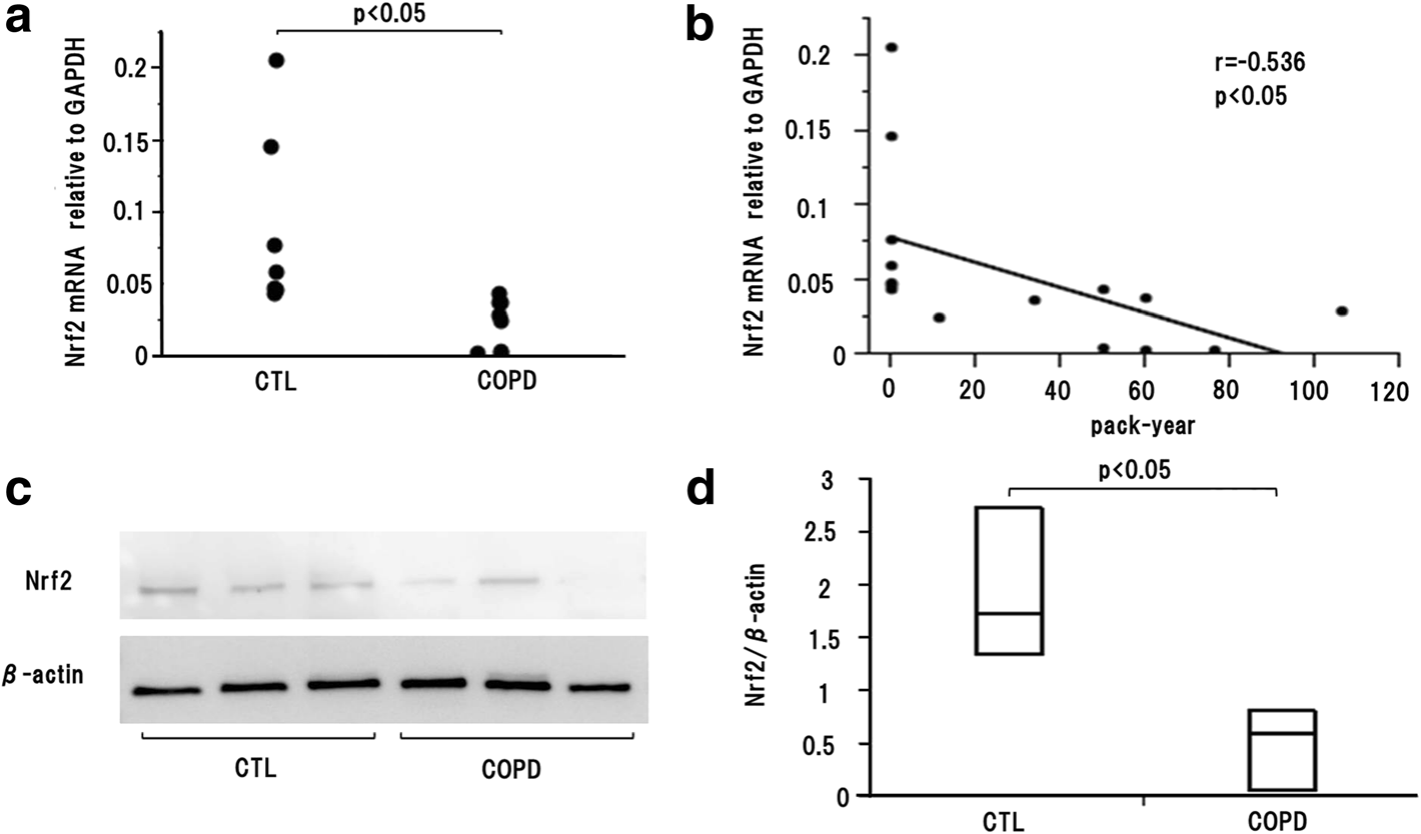
Nrf2 expression in study subjects. **a** Nrf2 mRNA expression in COPD subjects were significantly decreased compared with CTL subjects. **b** Nrf2 mRNA expression was significantly negatively correlated with pack-year. **c** Western blotting analysis of Nrf2 in bronchial epithelial cells in COPD and CTL subjects. **d** The blotting were normalized to β-actin and measured by densitometry. Nrf2 protein expression in COPD subjects were significantly decreased compared with CTL subjects. CTL: control

**Fig. 2:**
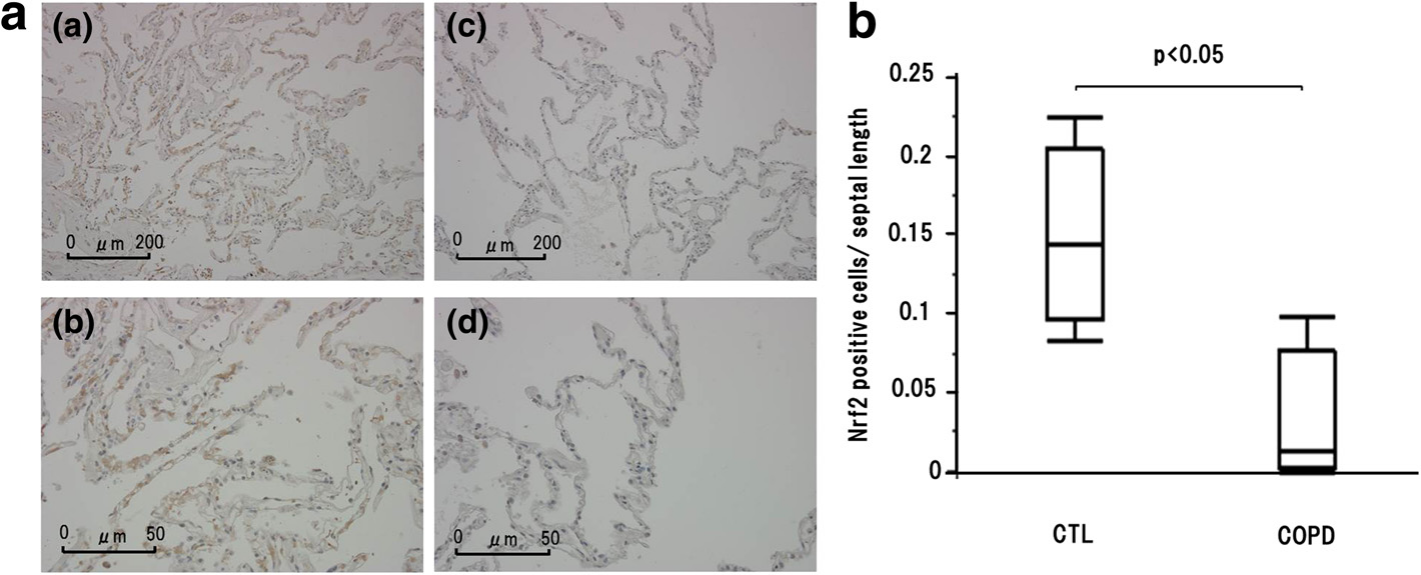
Nrf2 immunohistochemistry. **a** Immunohistochemical assessment of Nrf2. (*a*) CTL, x100 magnification, (*b*) CTL, x400 magnification, (*c*) COPD, x100 magnification and (*d*) COPD, x400 magnification. **b** Nrf2 expression in COPD subjects were significantly decreased compared with CTL in immunohisotochemical assessment. CTL: control

Subject characteristics were shown in Table 1. There were no significant differences in age or BMI. FEV1.0 and % FEV1 in COPD subjects were significantly lower than those in the CTL group. Nrf2 mRNA level in COPD subjects was significantly lower than that in the CTL subjects (Fig. 1a). There was significant negative correlation between Nrf2 mRNA expression and pack-year (Fig. 1b). Nrf2 protein level in COPD subjects was also significantly lower than that in the CTL subjects (Fig. 1c and d). There were Nrf2 positive epithelial cells in alveolar septum in both COPD and CTL subjects (Fig. 2a). In addition, the ratio of positive cells to alveolar septal length in COPD subjects was significantly decreased compared with CTL subjects (Fig. 2b).

**Table 1:** Subjects, characteristics

	Control	COPD	*p*-value
Subject No.	7	8	N.S.
Sex (male/female)	(2/5)	(6/2)	
Age (year)	62.7 (56.9–68.5)	70.0 (64.6–75.5)	N.S.
BMI (kg/m^2^)*	22.4 (19.1–25.8)	21.2 (18.1–24.3)	N.S.
Pack-Year*	0	55.9** (40.1–71.6)	*p* < 0.01
FEV1.0 (L)*	2.40 (1.89–2.90)	1.40** (1.00–1.79)	*p* < 0.01
% FEV1.0 (%)*	88.9 (73.4–104.4)	55.0 ** (42.8–67.3)	*p* < 0.01

*mean (95 % confidence interval) **p < 0.05 compared with control

BMI: body mass index

FEV1.0: Forced expiratory volume in one second

% FEV1.0: percent predicted forced expiratory volume in one second

### EBC pH decreased after cigarette smoking

**Fig. 3:**
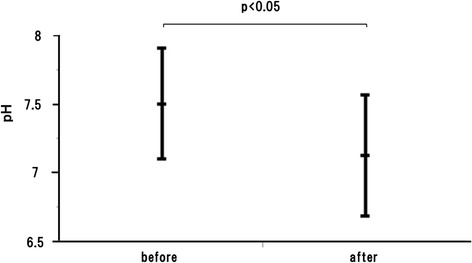
The pH of exhaled breath condensate before and after smoking. The pH of exhaled breath condensate (EBC) before and after smoking in non-COPD smoking subjects. The pH of EBC was significantly decreased after smoking. Data were expressed as Mean ± SEM

We measured the EBC pH in non-COPD smokers before and after cigarette smoking. The first EBC was collected after overnight non-smoking. They were then allowed to smoke 1 cigarette. After 15 min of smoking, the second EBC was collected. EBC pH was significantly lower after smoking (Fig. 3). These results indicate that smoking only 1 cigarette can cause oxidative stress in the airway. To mimic the oxidative milieu in smokers’ airways in in vitro experiments, we measured the pH of various concentrations of H_2_O_2_ and CSE diluted in PBS or culture medium. The pH of 100 μM H_2_O_2_ was 7.54. The pH of 10 and 15 % CSE were 7.75 and 7.56, respectively. We chose 10 and 15 % CSE as treatments and 100 μM H_2_O_2_ as a positive control in in vitro experiments.

### CSE-induced epithelial cell apoptosis

**Fig. 4:**
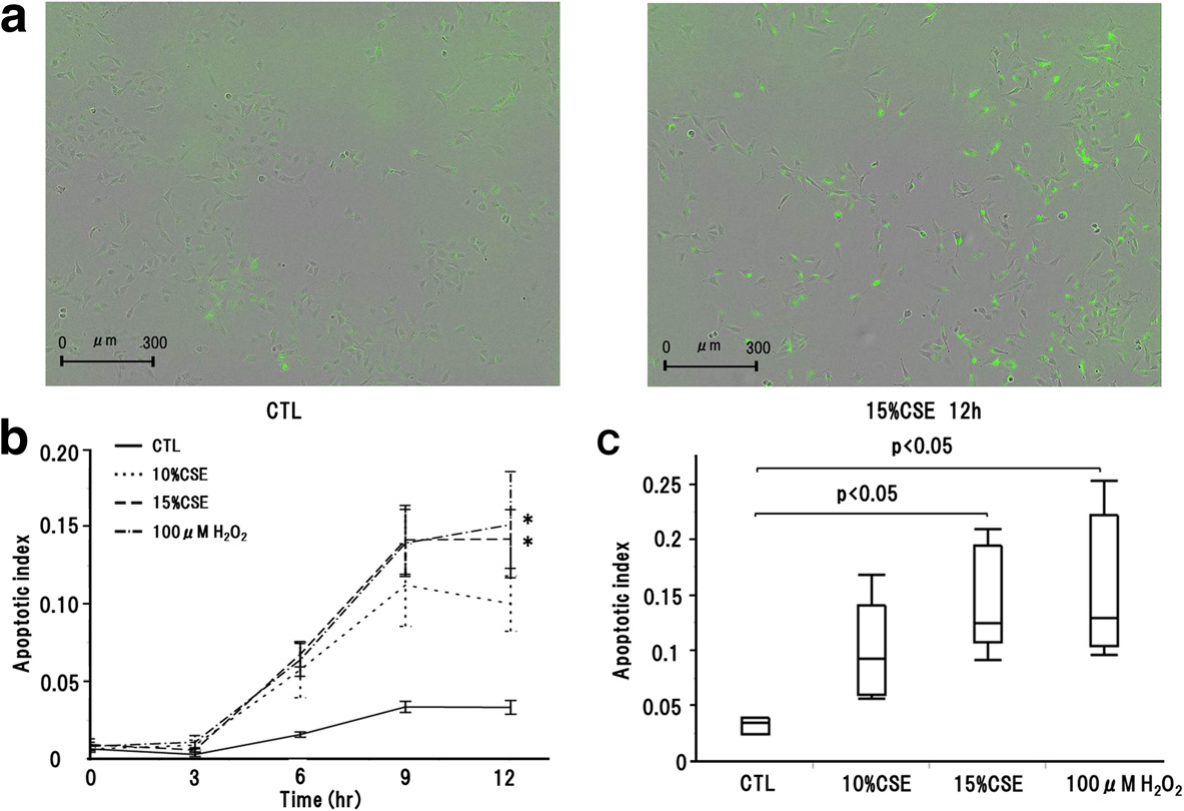
CSE induced apoptosis. **a** Green fluorescent staining by caspase-3/7 reagent revealed apoptosis cells. The number of green fluorescent staining was increased in 15 % CSE treatment. **b** Apoptotic index was increased over time in 10 % CSE, 15 % CSE or 100 μM H_2_O_2_ treatment compared with CTL. *: *p* < 0.05 compared with CTL. **c** Apoptotic index increased depending on CSE concentration. Apoptotic indices in 15 % CSE, 100 μM H_2_O_2_ treatment were significantly increased compared with CTL. CTL: control

**Fig. 5:**
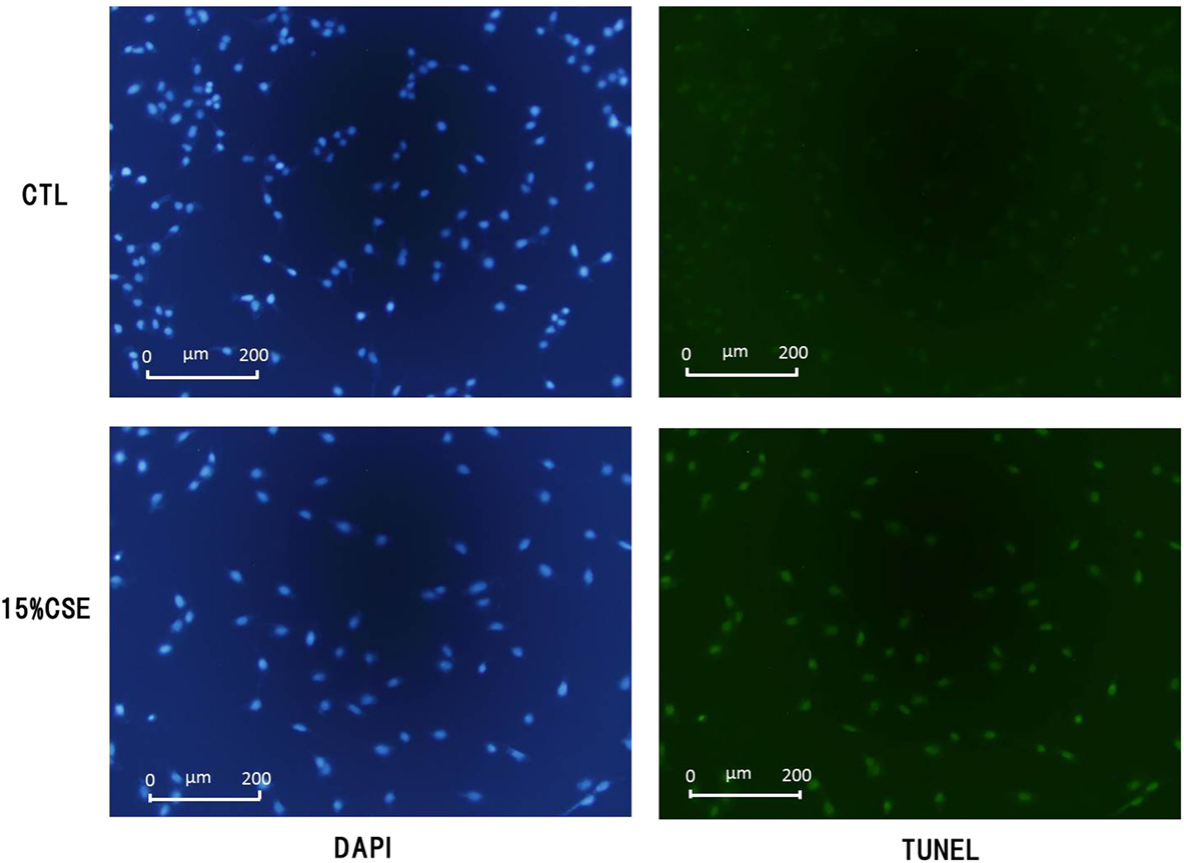
TUNEL stain in 15 % CSE treatment. DAPI (Left) and TUNEL (right) stain were performed on A549 cells after 24 h of 15 % CSE treatment or control. Nuclei were detected with DAPI fluorescence (blue) Apoptosis were detected with TUNEL fluorescence (green). Increased TUNEL fluorescence was observed in 15 % CSE treatment compared with control

Following 15 % CSE treatment, green fluorescent staining by caspase-3/7 reagent was higher than that in the control (Fig. 4a). The apoptotic index was time-dependent and the peak apoptotic index was recorded at 12 h after treatment (Fig. 4b). The apoptotic index also showed CSE concentration-dependence, and the apoptotic index of cells treated with 15 % CSE was significantly higher that of the control (Fig. 4c). To confirm CSE-induced apoptosis, we performed TUNEL staining in control and 15 % CSE-treated cells. TUNEL fluorescence was higher with 15 % CSE-treated than in the control cells (Fig. 5).

### Nrf2 knockdown increased CSE-induced epithelial cell apoptosis

**Fig. 6:**
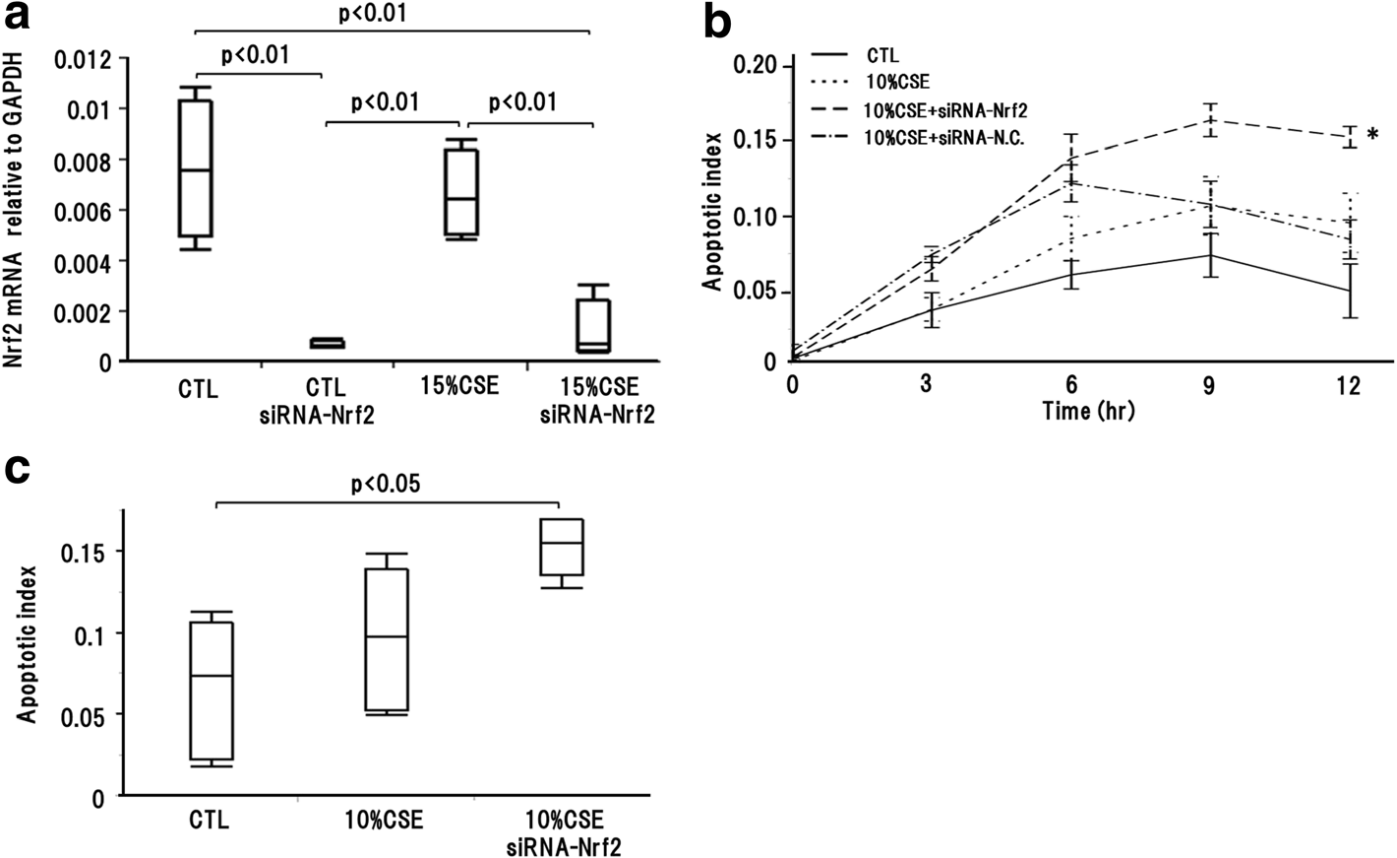
Effect of Nrf2 knock down on CSE induced apoptosis. **a** The siRNA-Nrf2 complex transfection with A549 cell significantly decreased Nrf2 mRNA expression in CTL and 15 % CSE. **b** Apoptotic index was increased over time in 10 % CSE or 10 % CSE with Nrf2 knockdown compared with CTL. *: *p* < 0.05 compared with CTL. **c** Apoptotic index was not significantly increased in 10 % CSE compared with CTL, while significantly increased in 10 % CSE with Nrf2 knockdown compared with CTL. CTL: control

Nrf2 is a central transcription factor for the antioxidant defense system. Oxidative stress causes apoptosis; therefore, we examined the effect of Nrf2 knockdown on apoptosis of A549 cells. A549 cells transfected with Nrf2 siRNA complex showed significantly decreased Nrf2 mRNA level in control conditions. Further, 15 % CSE treated A549 cells transfected with Nrf2 siRNA complex also showed significantly decreased Nrf2 mRNA level (Fig. 6a), mimicking cigarette smoke-associated COPD airways. 10 % CSE treatment condition and 10 % CSE treatment with Nrf2 mRNA knockdown increased apoptotic index over time (Fig. 6b). Althugh apoptotic index in 10 % CSE treatment was greater than that in control conditions, the difference between the indices did not reach statistical significance. However, the apoptotic index in 10 % CSE with Nrf2 mRNA knockdown was significantly greater than that in control conditions (Fig. 6c). These results suggested that decreased Nrf2 mRNA expression increased CSE-induced apoptosis of A549 cells.

### NAC attenuated CSE-induced epithelial cell apoptosis

**Fig. 7:**
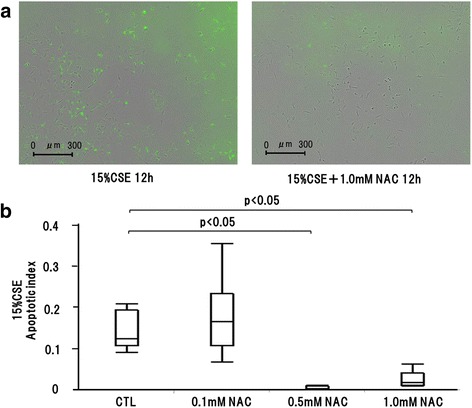
Effect of NAC pretreatment on CSE induced apoptosis. **a** Green fluorescent staining by caspase-3/7 reagent revealed apoptosis cells. The number of green fluorescent staining was decreased in 15 % CSE with 1.0 mM NAC pretreatment. **b** Pretreatment with 0.1 mM NAC did not decrease 15 % CSE induced apoptotic index, whereas pretreatment with 0.5, 1.0 mM NAC significantly decreased 15 % CSE induced apoptotic index

We investigated whether NAC protected against CSE-induced epithelial cell apoptosis. Green fluorescent staining by caspase-3/7 reagent in 1.0 μM NAC-pretreated 15 % CSE cells was lower than that in 15 % CSE-treated cells (Fig. 7a). Although pretreatment with 0.1 μM NAC did not reduce the 15 % CSE-induced apoptotic index, pretreatment with 0.5 or 1.0 μM NAC significantly reduced the 15 % CSE-induced apoptotic index (Fig. 7b). In contrast, pretreatment with 0.1, 1.0, or 5.0 μg/ml anti-TNF-α antibody did not reduce the apoptotic index of 10 or 15 % CSE-treated cells or of 100 μM H_2_O_2_-treated cells (data not shown). These findings suggest that CSE-induced apoptosis is caused by oxidative stress that is not mediated by the TNF-α receptor pathway.

## Discussion

In the present study, we demonstrated that Nrf2 expression in bronchial epithelial cells in COPD subjects was significantly lower than that in control subjects. In addition, we immunohistochemically showed that Nrf2 expression in alveolar epithelial cells was significantly lower than that in control subjects. Nrf2 plays an important role as the master regulator of antioxidant responses [13]. Nrf2 is constitutively expressed in almost all cell types and tissues, and is most abundant in tissues where detoxification reactions occur routinely, including the lungs. However, Nrf2 is also inducible and triggered by oxidative stress, injury, and inflammation. Cigarette smoke contains a substantial ROS, and exposure to cigarette smoke is known to induce transient Nrf2 expression in human airway cells. Upon stimulation, cytosolic Nrf2 reaches the nucleus and binds to ARE in the upstream promoter region of antioxidant genes and initiates transcription [4]. Among these, the glutathione cysteine ligase catalytic subunit (GCLC) and glutathione cysteine ligase modifier subunit (GCLM) are important for antioxidant defense. Nrf2 is required for the constitutive and inducible expression of GCLC and GCLM, which are required for glutathione (GSH) synthesis [14]. Impaired Nrf2 expression in COPD patients might fail to evoke an appropriate antioxidant response and allow excessive apoptosis resulting in lung tissue destruction and emphysema. A previous cohort study showed that certain polymorphisms of the Nrf2 gene correlated with accelerated limitations in airflow of smokers [15, 16]. Subjects with impaired Nrf2 might be related to COPD.

Cigarette smoke causes oxidative stress, enhances inflammation, inactivates critical anti-proteinase inhibitors such as α1-antitrypsin [17], and causes apoptosis of alveolar cells [18]. To determine the appropriate conditions for in vitro CSE study, we used the pH of EBC as a reference. Collection of EBC is a non-invasive and repeatable technique for monitoring the airway milieu. EBC pH is known to correlate with oxidative stress and H_2_O_2_ concentration [19]. A previous report observed that the mean EBC pH in patients with COPD and non-COPD smokers was significantly lower than that in non-smoking subjects [20]. We measured EBC pH before and after smoking and found that the EBC pH was immediately lowered after smoking. Our results confirmed that cigarette smoking caused oxidative stress in the human airway. We chose 10 % and 15 % CSE as treatments and 100 μM H_2_O_2_ as a positive control in in vitro CSE experiments. We determined the effect of oxidative stress by CSE on apoptosis over time using IncuCyte ZOOM and showed that the apoptotic index increased significantly at 12 h after treatment with 10 % and 15 % CSE. Because the doubling time of A549 cell is about 22 h and in order to avoid influence of cell division and growth, we adopted a 12-h time point for evaluation. Although we used caspase-3/7 activation as an indicator of apoptosis, we performed TUNEL staining to confirm apoptosis. The apoptosis index of A549 cells increased in a CSE dose-dependent manner. This is consistent with other in vitro studies in BEAS-2B cells [21]. However, alveolar epithelial cells are robustly involved in the pathogenesis of emphysema through the alveolar wall destruction and/or the loss due to epithelial cells apoptosis [22]. Thus, in this study we used A549 cells, alveolar epithelial cell line, instead of BEAS-2B cells.

Nrf2 is thought to be a key determinant of COPD susceptibly [23–26]. We examined the effect of Nrf2 knockdown on CSE-induced apoptosis. Nrf2 knockdown significantly increased 10 % CSE-induced apoptosis. In previous animal studies, Nrf2−/− mice were reported to be highly susceptible to cigarette smoke-induced lung injury [5, 6]. On the other hand, Nrf2 overexpression protected from cell apoptosis by cigarette smoke exposure [27, 28]. The present results support the idea that impaired Nrf2 expression plays an important role in apoptosis. We also explored the mechanism behind CSE-induced apoptosis. Caspase activation commits cells to one of two distinct but convergent pathways: the death receptor pathway and the mitochondrial pathway. The death receptor pathway is activated when members of the TNF-α superfamily bind to cell surface “death receptor” members of the TNF-α receptor family. Initiators of the mitochondrial pathway increase intracellular ROS, DNA damage, the unfolded protein response, and the deprivation of growth factor [29]. In the present study, we showed that NAC ameliorates CSE-induced apoptosis. However, anti-TNF-α antibody could not ameliorate CSE-induced apoptosis. CSE-induced apoptosis of A549 cells was dependent on the mitochondrial pathway following oxidative stress and independent of the death receptor pathway in this in vitro setting. NAC is a precursor of GSH, which is one of the most important antioxidants for protecting cells and tissue from ROS. GSH and NAC act as oxygen free radical scavengers [30]. GCLC, GCLM, and glutathione reductase (GR) are also critical for GSH. Our results showed that oxidative stress induced by CSE played a key role in apoptosis. Imbalance between oxidants and antioxidants is thought to be involved in the development of pulmonary emphysema [31, 32]. Thus, oxidative stress is an important therapeutic target, and NAC is thought to be a potential drug for COPD [33–36]. Although the BRONCUS study showed that NAC is ineffective at preventing COPD exacerbation in patients [37], other reports have shown that high-dose NAC significantly reduced COPD exacerbations [38, 39]. Therapies targeting oxidative stress are gradually showing more promising outcomes. The limitations of this study are: (1) small number of participants and (2) cell source (we collected epithelial cells around the sixth branch, not the alveolar region, for technical reasons). However, the present study is an important and extensive investigation into the relationship between Nrf2 and oxidative stress in airway compartments of patients with COPD.

## Conclusions

We demonstrated that Nrf2 expression in patients with COPD was lower than that in control subjects, suggesting that impaired Nrf2 expression might be related to COPD. Further research into the molecular mechanisms involved in Nrf2 expression in COPD airways and therapies to upregulate Nrf2 are needed. Administration of antioxidants to patients with COPD may be a basic therapeutic option to consider in addition to the current bronchodilator therapy.

## Abbreviations

ARE: Antioxidant response element
COPD: Chronic obstructive pulmonary disease
CSE: Cigarette smoke extracts
GCLC: Glutathione cysteine ligase catalytic subunit
GCLM: Glutathione cysteine ligase modifier subunit
NAC: N-acetyl cysteine
Nrf2: Nuclear factor erythroid 2-related factor 2
ROS: Reactive oxygen species
